# Flexible, Brain-Inspired Communication in Massive Wireless Networks

**DOI:** 10.3390/s20061587

**Published:** 2020-03-12

**Authors:** Bartosz Bossy, Pawel Kryszkiewicz, Hanna Bogucka

**Affiliations:** Department of Wireless Communications, Poznan University of Technology, 61-131 Poznan, Poland; pawel.kryszkiewicz@put.poznan.pl (P.K.); hanna.bogucka@put.poznan.pl (H.B.)

**Keywords:** brain inspiration, energy efficiency, analog and digital transmission, power consumption, massive communications, Internet of Things

## Abstract

In this paper, a new perspective of using flexible, brain-inspired, analog and digital wireless transmission in massive future networks, is presented. Inspired by the nervous impulses transmission mechanisms in the human brain which is highly energy efficient, we consider flexible, wireless analog and digital transmission on very short distances approached from the energy efficiency point of view. The energy efficiency metric is compared for the available transmission modes, taking the circuit power consumption model into account. In order to compare the considered systems, we assume that the transmitted data comes from analog sensors. In the case of the digital transmission scheme, the decoded data are converted back to analog form at the receiving side. Moreover, different power consumption models from the literature and the digital transmission schemes with different performance are analyzed in order to examine if, for some applications and for some channel conditions, the analog transmission can be the energy-efficient alternative of digital communication. The simulation results show that there exist some cases when the analog or simplified digital communication is more energy efficient than digital transmission with QAM modulation.

## 1. Introduction

In recent years, the number of devices connected to the Internet has rapidly increased. Mobile data traffic has grown 17-fold over the past five years [1]. Mobile networks carried 686 petabytes per month in 2012 [1]. According to Cisco predictions that monthly global mobile data traffic will be 77 exabytes by 2022, and annual traffic will reach almost one zettabyte [1]. Moreover, communication of billions of machines and devices that are expected to comprise the Internet of Things (IoT) pose even greater challenges, never encountered before. This means a high density of devices per square meter, and in consequence, short distances between nodes of the network. Furthermore, some number of these devices, e.g., sensors, will be (are) battery powered, and thus, they have to be ultra-energy-efficient. Nowadays, most low-energy wireless systems adopt digital modulation and coding, and the baseband digital signal processing is often performed in software [2]. Thus, besides the energy needed for signal transmission, energy is also required for signal processing at both a transmitter and a receiver. This processing energy may even dominate at short link distances and most often increases when approaching the Shannon capacity limit because of the implementation complexity [3].

The inspiration for the design of new ultra-energy-efficient networks can be the human brain which can be compared to the ultra-dense network. The neurons of a human brain can be compared to nodes, while axons can be compared to links in a network. It is well-known that the current wireless-node power-consumption in wireless local-area and cellular networks is of the order of 0.1–2 W [4] for just one transmission link. In comparison, the incredibly complex human brain works with less than $10^{-9}\;W$ per node (neuron) with up to 10,000 links. Thus, even considering the shorter distances, the human brain for a massive network of neurons and synapses is many orders of magnitude more energy efficient than any human-made network. We believe that future IoT networks should borrow some mechanisms used in the nervous-system communication, since the number of nodes will be massive, and the distances and power levels will be extremely small. One mechanism in a human brain, making it ultra-energy-efficient, is the fact that neurons operate and communicate continuously in time. Moreover, inside each neuron, information incoming from the synapses, is integrated and processed in an analog manner and the neuron then decides whether to fire an action potential. This discrete, binary event is transmitted along the axon to other neurons. Thus, we pose the question whether in some energy-constricted, dense networks (with short-distance links and moderate distortions), the nervous-system-inspired analog transmission can be an alternative to digital transmission, whether adaptive digital and analog modulation is an energy-efficient link-adaptation option, and if so, in what network scenarios.

Bio-inspired systems have been studied intensively during the last 15 years, as can be seen from the review papers, see for example [5,6,7,8]. Nevertheless, these papers describe the general concept of bio-inspired systems or summarize the bio-inspired algorithms, e.g., genetic algorithm or ant-colony optimization. In the context of wireless networks, there are few articles that present specific, brain-inspired solutions. For instance, in [9], the authors describe the topologies of a network which can be found in a human brain and show the trade-off between the complexity of the topology and the consumed power. The brain-inspired dynamic spectrum management for cognitive radio has been proposed in [10]. Papers [11,12] describe the possibility of applying the artificial intelligence (AI) techniques (like machine learning, genetic algorithms, swarm intelligence, ant colony, artificial neural networks, fuzzy system) in the context of 5G networks. Moreover, in [13] the microglia functionality from the human brain is applied in a dense network. It was already shown in [14] that the digital system with coding can be more energy efficient for longer links while the uncoded digital system is more energy efficient for shorter links. We wanted to extend this research to an analog system that requires higher transmission power (to provide higher SNR at the receiver as confirmed by Figure 6), but lower power is consumed by signal processing. Finding the balance between these two factors and its relation to coded and uncoded digital systems is a novel research topic up to our knowledge.

In this paper, we consider brain-inspired analog and digital transmission in the context of energy-efficient ultra-dense networks, i.e., consisting of short links with moderate attenuation and distortions. The energy-efficiency metric has been compared between these two types of communication taking the circuit power consumption model into account. Moreover, we assumed that the transmitted data came from analog sensors in the considered systems. Although currently many sensors are available as digital output only, a number of physical phenomenons are measured initially as analog signals, e.g., voltage or resistance. Therefore, in the case of the digital transmission, the input data are fed first to the analog-to-digital converter. At the receiving side, the decoded data are converted back to analog form. The rest of the paper is organized as follows. In Section 2, the system model for analog and digital transmission is described. The numerical results with their detailed analysis are presented in Section 3, while the conclusions are provided in Section 5.

## 2. System Model

**Figure 1:**
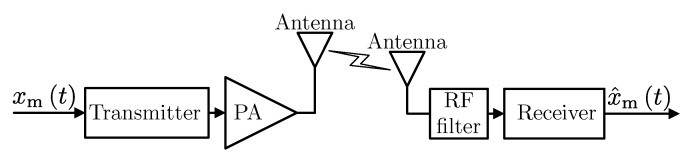
The general block diagram of the considered system.

Let us consider the single wireless link presented in Figure 1. The analog source (modulating) signal $x_{m}\left(t\right)$ is processed in the transmitter (either digital or analog), and then, the signal at the radio frequency is amplified in the power amplifier (PA) and fed to the antenna. In the receiver, the opposite operations are performed on the signal received by the antenna. It is assumed that the wireless channel introduces additive, white Gaussian noise as a distortion. Therefore, channel equalization at the receiver is not considered. This simplifies the considered model and is a reasonable assumption in IoT systems that are typically narrowband. In order to determine the quality of wireless transmission (both analog and digital), receiver’s analog output ${\hat{x}}_{m}\left(t\right)$ is compared with source signal $x_{m}\left(t\right)$ using the mean square error metric (MSE) defined as $E\left[{\left({\hat{x}}_{m}\left(t\right)-x_{m}\left(t\right)\right)}^{2}\right]$ where expectation can be calculated over time or random input signal realizations.

### 2.1. Analog Communication Power Consumption

**Figure 2:**
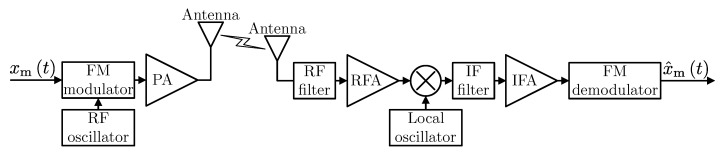
The transmitter and receiver block diagram of analog transmission schemes.

The high energy efficiency of a human brain results from the fact that inside each neuron, information incoming from synapses is integrated and processed in a simple, analog manner, thus avoiding additional energy-intensive processing. For our communication model, let us consider the well-known frequency modulation $\left(\mathrm{FM}\right)$ and analyze it from the energy-efficiency perspective. The block diagram of the considered FM system is presented in Figure 2. For modulating signal $x_{m}\left(t\right)$ and carrier wave $x_{c}\left(t\right)=A_{c}\cos\left(2\pi f_{c}t\right)$ (where $f_{c}$ is the carrier’s base frequency, and $A_{c}$ is the carrier’s amplitude), the modulated signal has the following form [15]:

(1) $$\begin{array}{c} x_{\mathrm{FM}}\left(t\right)=A_{c}\cos\left(2\pi f_{c}t+2\pi \Delta f\int_{0}^{t}x_{m}\left(\tau \right)d\tau \right), \end{array}$$

where Δf is the frequency deviation. The Carson’s rule defines the bandwidth wherein about 98% of the power of a frequency-modulated signal lies:

(2) $$\begin{array}{c} B_{\mathrm{sigA}}=2\left(\Delta f+f_{m}\right), \end{array}$$

where $f_{m}$ is the highest frequency component of $x_{m}\left(t\right)$.

In order to analyze the energy efficiency of the considered analog transmission, it is necessary to estimate the total power consumed, which results from the structure of the transmitter and receiver. For our transmitter and receiver model presented in Figure 2, the total power consumption can be calculated as:

(3) $$\begin{array}{cc} P_{\mathrm{totA}} & =P_{\mathrm{PA}}+P_{C}+P_{\mathrm{FMmod}}+P_{\mathrm{FMdemod}}, \end{array}$$

where $P_{\mathrm{PA}}$ is the power consumed by the power amplifier, while $P_{C}$ is the power required to run the circuit components such as oscillators, mixers, filters, radio and intermediate frequency amplifiers (RFA, IFA). Moreover, the powers consumed by the FM modulator and demodulator are denoted as: $P_{\mathrm{FMmod}}$, $P_{\mathrm{FMdemod}}$. The direct FM modulation can be implemented using a Voltage-Controlled Oscillator (VCO). In [16], the power consumption model of VCO based on a resonant circuit is presented. Based on Equation (7) in [16], it can be observed that the power consumption of VCO is independent on dynamically changing transmission parameters and the authors assumed the constant value. In the case of demodulation of FM signals, which can be realized by the Phase Locked Loop (PLL), the situation is similar. In [16,17], the authors proposed the PLL power consumption model, where the power consumption of PLL takes the constant value. Based on the power consumption models described in [3,14,16,18], $P_{C}$ can be modeled as the constant value as well, while the power consumption of PA class B as [19]:

(4) $$\begin{array}{c} P_{\mathrm{PA}}=\frac{4}{\pi }\sqrt{P_{\mathrm{TX}}\cdot PAR\cdot P_{T}\left(t\right)}, \end{array}$$

where $P_{\mathrm{TX}}$ is the average transmission power, PAR determines Peak to Average Power Ratio while $P_{T}\left(t\right)$ is the transmission power at time *t*. Thus, the power consumption model from Equation (3) can be simplified to:

(5) $$\begin{array}{cc} P_{\mathrm{totA}} & =P_{\mathrm{PA}}+P_{\mathrm{CA}}, \end{array}$$

where $P_{\mathrm{CA}}=P_{C}+P_{\mathrm{FMmod}}+P_{\mathrm{FMdemod}}$. The power consumption model presented above is relatively simple but indeed realistic and based on models from literature. Moreover, in Table 1, the power consumption values of the FM chips are given. It can be observed that the consumed power highly depends on hardware implementation and applications, and the presented model can be easily scaled.

### 2.2. Digital Communication Power Consumption Model

**Figure 3:**
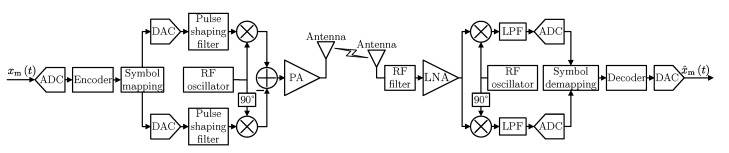
The transmitter and receiver block diagram of digital transmission schemes with QAM modulation.

Let us now consider digital transmissions system with Quadrature Amplitude Modulation (QAM) presented in Figure 3, where the analog input signal $x_{m}\left(t\right)$ is sampled with sampling frequency $f_{s}$ and quantized using $N_{b}$ bits. Then, the data in the digital form are processed in the forward error correction (FEC) encoder, mapped to QAM symbols, which modulate the complex carrier. For the signal at the output of the digital to analog converters (DAC), the pulse is shaped using the raised-cosine pulse shaping filter with roll-off factor β. Finally, the signal converted to the radio frequency is amplified in PA and fed to the antenna. These processes have to be inverted at the receiver in order to get the estimate of the original signal. The bandwidth of the transmitted signal can be calculated as:

(6) $$\begin{array}{c} B_{\mathrm{sigD}}=\frac{\left(1+\beta \right)R_{s}}{2}, \end{array}$$

where $R_{s}$ is the symbol rate of the transmitted signal, and is given by:

(7) $$\begin{array}{c} R_{s}=\frac{f_{s}N_{b}}{R_{\mathrm{cod}}\log_{2}\left(M\right)}. \end{array}$$

In the above equation, $R_{\mathrm{cod}}$ determines the code rate (in the case of an uncoded scheme $R_{\mathrm{cod}}=1$), and *M* is the QAM constellation order.

**Figure 4:**
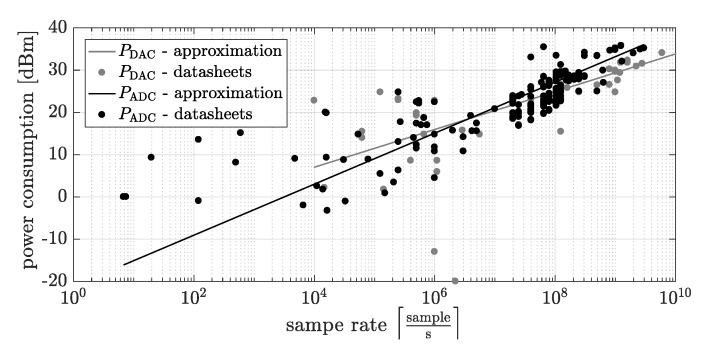
The powers consumed by the ADC and DAC based on datasheets of Analog Devices chips.

The power consumption of the digital transmission is very difficult to estimate due to the great dependence of the implementation and hardware performance. Nevertheless, in the literature, there exist high-level power consumption models, which can be used to analyze the energy efficiency of the considered system. The most popular and general model assumes that the power dissipation in a chip can be modeled as the sum of a static term and a dynamic term [20]:

(8) $$\begin{array}{c} P_{\mathrm{totD}}^{\mathrm{QAM}}=P_{\mathrm{CD}}^{\mathrm{QAM}}+P_{\mathrm{PA}}+2P_{\mathrm{ADC}}+2P_{\mathrm{DAC}}+P_{\mathrm{COMP}}, \end{array}$$

where $P_{\mathrm{CD}}^{\mathrm{QAM}}$ is the power consumed by the transmitter and receiver active components such as mixers, filters, oscillators, modulator and demodulator, $P_{\mathrm{ADC}}$, $P_{\mathrm{DAC}}$ describe the powers consumption of the analog to digital converter (ADC) and DAC, respectively. Most importantly, the two-channel converters are assumed, so that event though 3 DACs and 3 ADCs are visible in Figure 3, the power consumption of each DAC/ADC is multiplied by 2 in Equation (8). The last term, $P_{\mathrm{COMP}}$, determines the power consumption of the baseband signal processing including encoding, decoding, symbol mapping, demapping, channel estimation and correction, etc.). The powers consumed by the ADC and DAC have been extracted from $P_{\mathrm{CD}}^{\mathrm{QAM}}$ because they depend on the sample rate being a changeable parameter in our system. Based on datasheets of the ADC and DAC produced by Analog Devices, the power consumption model has been proposed. Form our analysis, it turns out that the consumed power mainly depends on the number of channels, the device architecture and the sample rate $R_{\mathrm{samp}}$ of the ADC and DAC and not on the number of bits as in the power consumption models in [14,16,18]. Based on real values of power consumption of $P_{\mathrm{ADC}}$ and $P_{\mathrm{DAC}}$ presented in Figure 4, the power consumption model of the converters have been proposed using *Curve Fitting Toolbox* being part of MATLAB software. The approximations of the power consumption of the ADC and DAC as a function of $f_{s}$ (presented in Figure 4) are given by: $P_{\mathrm{ADC}}=7.719\cdot 10^{-6}{f_{s}}^{0.6036}$ and $P_{\mathrm{DAC}}=8.219\cdot 10^{-5}{f_{s}}^{0.447}$, respectively.

The power consumption of the signal processing (e.g., encoding, decoding, the channel estimation process) is usually modeled as $P_{\mathrm{COMP}}=\xi R$, where *R* is the achieved link-throughput, while ξ is the computational efficiency in $W/\left(\mathrm{bit}/s\right)$. This relatively simple model not only has been applied in many papers focusing on the energy efficiency optimization [21,22,23], but it has also been confirmed by measurements in [4]. In the case of $P_{\mathrm{PA}}$, we assume the same class and the same power consumption model as in Section 2.1.

**Figure 5:**
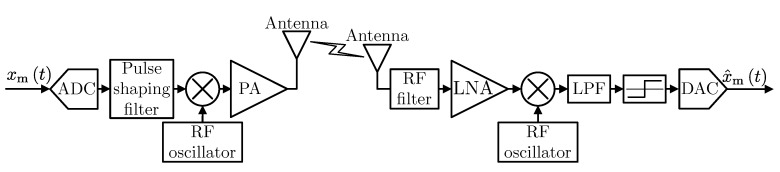
The transmitter and receiver block diagram of uncoded digital ASK modulation system.

Moreover, for the purpose of diverse comparisons, let us also consider a simpler digital modulation scheme, such as uncoded Amplitude-Shift Keying (ASK) modulation, which is commonly used in key fobs or in devices for controlling other electronics. The block diagram of the transmitter and receiver using ASK modulation is presented in Figure 5. It can be observed that the input signal is sampled with $f_{s}$, quantized using $N_{b}$ bits, and the signal at the output of DCA is shaped in the raised-cosine pulse shaping filter with roll-off factor β. Thus, the occupied bandwidth for the ASK modulation, $B_{\mathrm{sigD}}^{\mathrm{ASK}}$, can be calculated by Equation (6) for M=2 and $R_{\mathrm{cod}}=1$. Intuitively, digital communication with ASK modulation (the block diagram shown in Figure 5) can be more energy efficient than the QAM-based encoded system (the one from Figure 3) due to a smaller number of components. On the other hand, the transmission power required to achieve a given quality can be higher.

The total consumption power of the digital communication with ASK modulation is given by:

(9) $$\begin{array}{c} P_{\mathrm{totD}}^{\mathrm{ASK}}=P_{\mathrm{CD}}^{\mathrm{ASK}}+P_{\mathrm{PA}}+P_{\mathrm{ADC}}+P_{\mathrm{DAC}}, \end{array}$$

where $P_{\mathrm{CD}}^{\mathrm{ASK}}$ is the power consumed by the transmitter and receiver components such as mixers, filters, oscillator, etc., and $P_{\mathrm{CD}}^{\mathrm{ASK}}\leq P_{\mathrm{CD}}^{\mathrm{QAM}}$.

## 3. Simulation Results

Although the analog communication schemes can potentially be energy-efficient because of a small number of the transmitter- and receiver-components, most of the wireless communication systems nowadays are digital for the reason of relatively high transmission quality performance, especially in the hostile radio channel. However, in some short-distance channels (such as the ones that can be potentially the case in massive high-density networks) the former can be competitive for the energy-efficiency with reasonable performance. Therefore, let us evaluate both transmission types in terms of the energy-efficiency and MSE-based performance.

Here below, results obtained by the computer simulation using MATLAB software are presented. It can be observed that in the case of the digital transmission, the input data are fed first to the analog-to-digital converter. At the receiving side, the decoded data are converted back to analog form. As the input and output signal of all systems has the same structure, their performance can be compared without consideration of the further processing steps and the energy consumed by them. Firstly, let us define the relation between the power consumed by the transmitter and receiver components in the above models. The hypothesis that analog communication can be more energy efficient than the digital is based on the assumption that the analog transmitter and receiver are less complex than digital ones. However, this also depends on the implementation and application. On the other hand, the FM or uncoded ASK transmitters and receivers use most of the blocks of the coded or uncoded digital system with QAM modulation. Thus, in our model, we introduce the factor $\zeta_{\mathrm{FM}}$ which determines the relation between $P_{\mathrm{CA}}$ and $P_{\mathrm{CD}}^{\mathrm{QAM}}$ where $P_{\mathrm{CA}}=\zeta_{\mathrm{FM}}P_{\mathrm{CD}}^{\mathrm{QAM}}$. The same operation can be applied in the case of the digital system with ASK modulation $P_{\mathrm{CD}}^{\mathrm{ASK}}=\zeta_{\mathrm{ASK}}P_{\mathrm{CD}}^{\mathrm{QAM}}$, where $\zeta_{\mathrm{ASK}}$ determines the relation between the power consumed by transmitter and receiver components in the digital transmission with ASK and QAM modulation. For the fair comparison of the analog and digital transmission in the considered scenario, both systems are compared for the same bandwidth $B_{\mathrm{sig}}=B_{\mathrm{sigA}}=B_{\mathrm{sigD}}=B_{\mathrm{sigD}}^{\mathrm{ASK}}$ and the same SNR in the transmission band. Thus, for the given parameters of digital transmission defining the bandwidth (code rate, bits number of quantization, modulation order and frequency sampling), the frequency deviation for analog transmission is determined. The results have been obtained for $f_{m}=15\;\mathrm{kHz}$, $f_{s}\;=\;44\;\mathrm{kHz}$, $f_{c}=3.5\;\mathrm{GHz}$, β=0.25, $N_{b}=\left\{9,10,11,12,13,14\right\}$, modulation orders for the QAM scheme $M=\left\{4,16,64,256\right\}$, the code rate of the turbo code for the coded transmission $R_{\mathrm{cod}}=1/3$, $P_{\mathrm{CD}}^{\mathrm{QAM}}=270\;\mathrm{mW}$ based on [16,24], ξ=0.9W/Mbit [25], $\zeta_{\mathrm{FM}}=\left\{1,0.75,0.5,0.25\right\}$, $\zeta_{\mathrm{ASK}}=\left\{1,0.8,0.5,0.25\right\}$, the values $\zeta_{\mathrm{FM}}=0.75$ and $\zeta_{\mathrm{ASK}}=0.8$ based on literature [16], channel attenuation $10\log_{10}\left({\left|h\right|}^{2}\right)\;=15\;-\;$ $\left(128.1+37.6\log_{10}\left(d\left[\mathrm{km}\right]\right)+21\log_{10}\left(\frac{f_{c}}{2\cdot 10^{9}}\right)\right)$ [26], the noise power $N=-174+10\log_{10}\left(B_{\mathrm{sig}}\right)+5\mathrm{dBm}$ [26]. In all systems, perfect synchronization is assumed. The results have been generated using the Monte Carlo method and have been averaged over 1000 source signal realization. The source signal has been generated as the sum of the sin function with different amplitude, frequency and phase. The time duration of input signal is equal 0.1 s.

**Figure 6:**
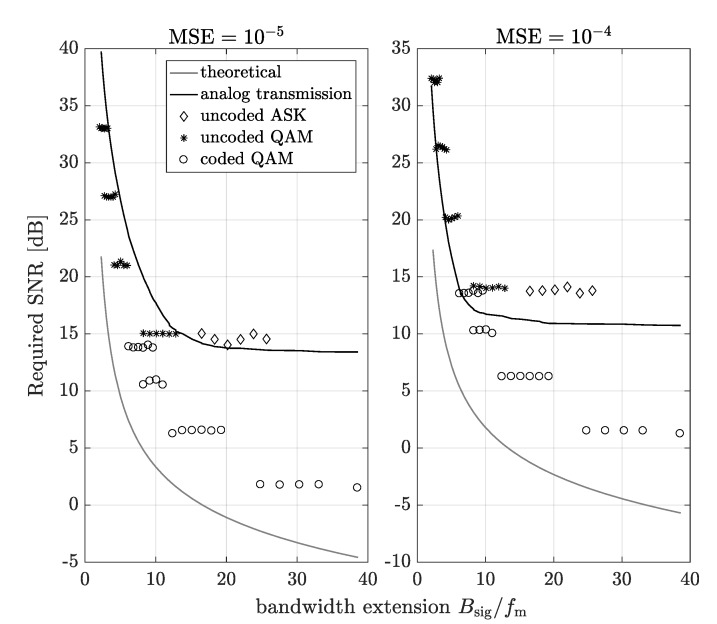
Required SNR needed to achieve $\mathrm{MSE}=10^{-5}$ (on the left) and $\mathrm{MSE}=10^{-4}$ (on the right) vs. the bandwidth extension, where $B_{\mathrm{sig}}=B_{\mathrm{sigA}}=B_{\mathrm{sigD}}=B_{\mathrm{sigD}}^{\mathrm{ASK}}$.

In the first step, the analog and digital transmission have been compared in the context of the SNR (corresponding to the transmit power) required to achieve a given quality of the received signal. Thus, in Figure 6, the required SNR needed to achieve a given MSE (defined in Section 2), as a function of the bandwidth extension for the analog, digital and the theoretical (ideal) scheme is presented. Based on [27], in the theoretical scheme, the required SNR is calculated assuming that the throughput of the link is equal to the link capacity for the extension bandwidth:

(10) $$\begin{array}{c} f_{m}\log_{2}\left(1+\frac{S}{\mathrm{MSE}}\right)=B_{\mathrm{sig}}\log_{2}\left(1+{\mathrm{SNR}}_{\mathrm{req}}\right), \end{array}$$

where *S* is a power of the analog source signal $x_{m}\left(t\right)$, while ${\mathrm{SNR}}_{\mathrm{req}}$ is the required value of SNR needed to achieve a given MSE, in the theoretical scheme. On the left-hand side of Figure 6, required SNR for $\mathrm{MSE}=10^{-5}$ is plotted. It is noticeable that in the most cases of the bandwidth extension, the digital transmissions achieve a given MSE for lower values of SNR than analog transmission. On the other hand, note that these results do not take the power consumed by the signal processing into account. Thus, from the energy efficiency perspective, the analog transmission may still be better. On the right-hand side of Figure 6, the required SNR for $\mathrm{MSE}=10^{-4}$ is plotted. There exist more cases where the analog transmission needs lower values of SNR, in order to achieve a given MSE.

**Figure 7:**
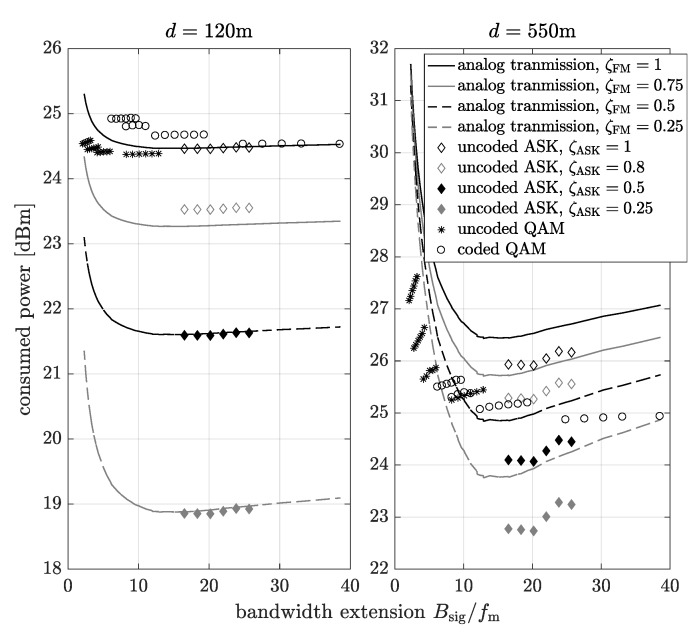
The power consumption vs. the bandwidth extension.

In Figure 7 the total consumed power as a function of the bandwidth extension for different distances between the transmitter and the receiver, as well as factors $\zeta_{\mathrm{FM}}$ and $\zeta_{\mathrm{ASK}}$ is plotted. Now, it can be observed that for a short distance between the transmitter and the receiver (d=120 m, on the left-hand side plot) the digital transmission can consume less power only when $\zeta_{\mathrm{FM}}=1$, i.e., when $P_{\mathrm{CD}}=P_{\mathrm{CA}}$ and even in this case, some of the considered digital transmission schemes consume more power than the analog transmission. Moreover, the uncoded digital transmission with ASK modulation can consume similar power as analog communication system, thus, it can be considered as a good candidate transmission scheme for energy efficient communication in short links. In the case of the higher distance the between transmitter and receiver (d=550 m, on the right-hand side plot), the analog transmission consumes more power than digital communication with QAM modulation for $\zeta_{\mathrm{FM}}\geq 0.75$, however, for $\zeta_{\mathrm{FM}}\leq 0.75$, there exist cases when the digital transmission is less energy efficient. Finally, for the considered analog as well as digital transmission, there exists the bandwidth extension which minimizes the consumed power.

**Figure 8:**
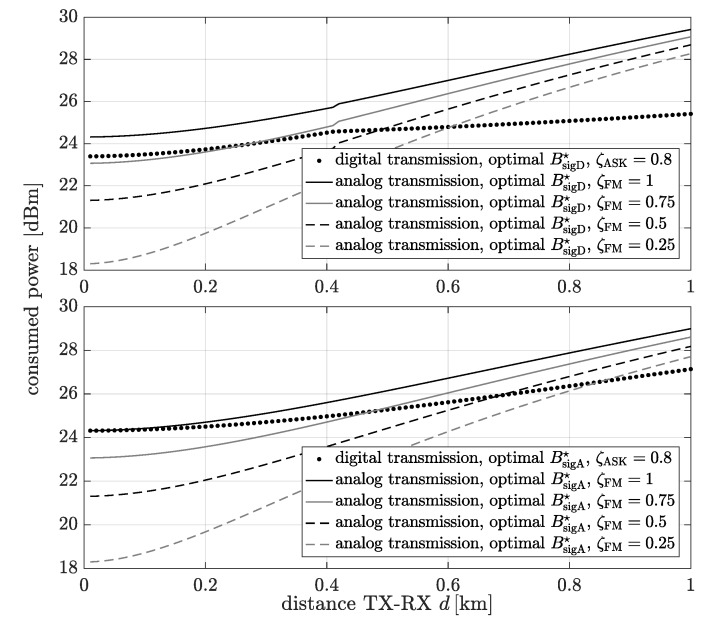
The consumed power as a function of the distance between transmitter and receiver for the bandwidth extension which minimize the power consumption of the digital scheme (top figure) and the analog scheme (bottom figure).

The power consumption of the analog and digital communication system schemes for the bandwidth extension which minimize the power consumption as a function of the distance between the transmitter and the receiver is shown in Figure 8. The digital communication system means that data can be transmitted using QAM or ASK modulation. The consumed power has been determined for each distance and the bandwidth extension which minimize the consumption power for digital transmission (top figure) $B_{\mathrm{sigD}}^{☆}$ or analog transmission (bottom figure) $B_{\mathrm{sigA}}^{☆}$ for $\mathrm{MSE}=10^{-5}$. It is noticeable that only when $\zeta_{\mathrm{FM}}=1$, the consumed power of the analog communication system is higher than the digital one for all considered distances. In the rest of the cases, for short distances, the analog transmission is more energy efficient. This is due to the domination of the power consumed by the transmitter and receiver components and the baseband signal processing power over the transmission (signal emission) power.

**Figure 9:**
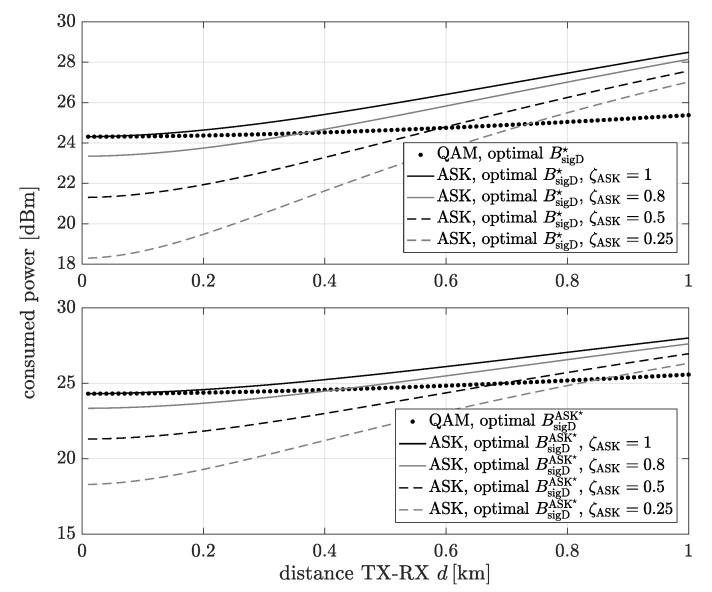
The power consumption for digital transmission with QAM and ASK modulation as a function of the distance between transmitter and receiver for $\mathrm{MSE}=10^{-5}$ for the bandwidth extension which minimize the power consumption of the digital transmission with QAM modulation (top figure) and the digital transmission with ASK modulation (bottom figure)

So far we have considered the source of the $x_{m}\left(t\right)$ signal to be time-continuous. Let us also consider the case, when the source generates digital data (discrete in time and in the set of values). For this case, in Figure 9, the power consumption of the digital transmission with QAM and ASK modulation as a function of the distance between the transmitter and the receiver is compared. In the top figure, the results have been determined for the optimal bandwidth extension (in the context of the minimization of power consumption) of the digital transmission with QAM modulation $B_{\mathrm{sigD}}^{☆}$, while in the bottom figure, for the optimal bandwidth extension (in the context of the minimization of power consumption) of the digital transmission with ASK modulation $B_{\mathrm{sigD}}^{{\mathrm{ASK}}^{☆}}$. There, one can observe that for short distances, the digital communication system with ASK modulation is more energy efficient than the one with QAM modulation due to the less complex transmitter and the receiver.

## 4. Analytical Power Consumption Model

In this section, the analytical power consumption model of the analyzed analog and digital transmission, based on the simulation results, have been proposed. Thus, let us approximate the required SNR for FM-based system vs. the bandwidth extension curves by the exponential function:

(11) $$\begin{array}{c} {\mathrm{SNR}}_{\mathrm{req}}^{\mathrm{FM}}\left[\mathrm{dB}\right]=a^{\mathrm{FM}}\exp\left(\frac{b^{\mathrm{FM}}B_{\mathrm{sig}}}{f_{m}}\right)+c^{\mathrm{FM}}\exp\left(\frac{d^{\mathrm{FM}}B_{\mathrm{sig}}}{f_{m}}\right), \end{array}$$

where $a_{\mathrm{FM}}$, $b_{\mathrm{FM}}$, $c_{\mathrm{FM}}$ and $d_{\mathrm{FM}}$ are the fitting parameters for a given MSE. The coefficients, obtained by least squares fitting to results visible in Figure 6, are shown in Table 2. Based on Equation (11), the transmission power for a given channel attenuation and the noise power can be calculated by:

(12) $$\begin{array}{c} P_{\mathrm{TX}}^{\mathrm{FM}}\left[\mathrm{dB}\right]={\mathrm{SNR}}_{\mathrm{req}}^{\mathrm{FM}}\left[\mathrm{dB}\right]-10\log_{10}\left({\left|h\right|}^{2}\right)+N\left[\mathrm{dB}\right]. \end{array}$$

Substituting Equation (12) to Equation (5), the analytical power consumption model which depends on the distance between transmitter and receiver and bandwidth extension can be determined:

(13) $$\begin{array}{cc} P_{\mathrm{totA}} & =\frac{4}{\pi }\sqrt{10^{\frac{P_{\mathrm{TX}}^{\mathrm{FM}}\left[\mathrm{dB}\right]}{10}}\cdot P_{T}\left(t\right)}+P_{\mathrm{CA}}. \end{array}$$

In the same way, the analytical power consumption model can be determined for the digital schemes. Due to the finite set of parameters, we determine the lower and upper bound for the required SNR in a function of bandwidth extension. The approximation of the required SNR for coded digital scheme can be defined by the exponential function:

(14) $$\begin{array}{c} {\mathrm{SNR}}_{\mathrm{req}}^{\mathrm{QAM}}\left[\mathrm{dB}\right]=a_{\mathrm{coded}}^{\mathrm{QAM}}\exp\left(\frac{b_{\mathrm{coded}}^{\mathrm{QAM}}B_{\mathrm{sig}}}{f_{m}}\right)+c_{\mathrm{coded}}^{\mathrm{QAM}}\exp\left(\frac{d_{\mathrm{coded}}^{\mathrm{QAM}}B_{\mathrm{sig}}}{f_{m}}\right), \end{array}$$

while for the uncoded QAM scheme by the function:

(15) $$\begin{array}{c} {\mathrm{SNR}}_{\mathrm{req}}^{\mathrm{QAM}}\left[\mathrm{dB}\right]=a_{\mathrm{uncoded}}^{\mathrm{QAM}}{\left(\frac{B_{\mathrm{sig}}}{f_{m}}\right)}^{b_{\mathrm{uncoded}}^{\mathrm{QAM}}}+c_{\mathrm{uncoded}}^{\mathrm{QAM}}, \end{array}$$

where the fitting parameters are $a_{\mathrm{coded}}^{\mathrm{QAM}}$, $b_{\mathrm{coded}}^{\mathrm{QAM}}$, $c_{\mathrm{coded}}^{\mathrm{QAM}}$, $d_{\mathrm{coded}}^{\mathrm{QAM}}$, $a_{\mathrm{uncoded}}^{\mathrm{QAM}}$, $b_{\mathrm{uncoded}}^{\mathrm{QAM}}$ and $c_{\mathrm{uncoded}}^{\mathrm{QAM}}$. These coefficient values, obtained based on least squares fitting to data from Figure 6, are given in Table 3 and Table 4 for coded and uncoded QAM system, respectively. Based on the above equations, the transmission power of digital scheme with QAM modulation can be calculated as:

(16) $$\begin{array}{c} P_{\mathrm{TX}}^{\mathrm{QAM}}\left[\mathrm{dB}\right]={\mathrm{SNR}}_{\mathrm{req}}^{\mathrm{QAM}}\left[\mathrm{dB}\right]-10\log_{10}\left({\left|h\right|}^{2}\right)+N\left[\mathrm{dB}\right]. \end{array}$$

Finally, substituting Equation (16) to Equation (8), the analytical power consumption model of the digital transmission with QAM modulation is obtained as:

(17) $$\begin{array}{c} P_{\mathrm{totD}}^{\mathrm{QAM}}=P_{\mathrm{CD}}^{\mathrm{QAM}}+\frac{4}{\pi }\sqrt{10^{\frac{P_{\mathrm{TX}}^{\mathrm{QAM}}\left[\mathrm{dB}\right]}{10}}\cdot PAR\cdot P_{T}\left(t\right)}+15.438\cdot 10^{-6}{f_{s}}^{0.6036}\\ +16.437\cdot 10^{-5}{f_{s}}^{0.447}+\xi B_{\mathrm{sig}}\log_{2}\left(1+10^{\frac{{\mathrm{SNR}}_{\mathrm{req}}^{\mathrm{QAM}}\left[\mathrm{dB}\right]}{10}}\right). \end{array}$$

**Figure 10:**
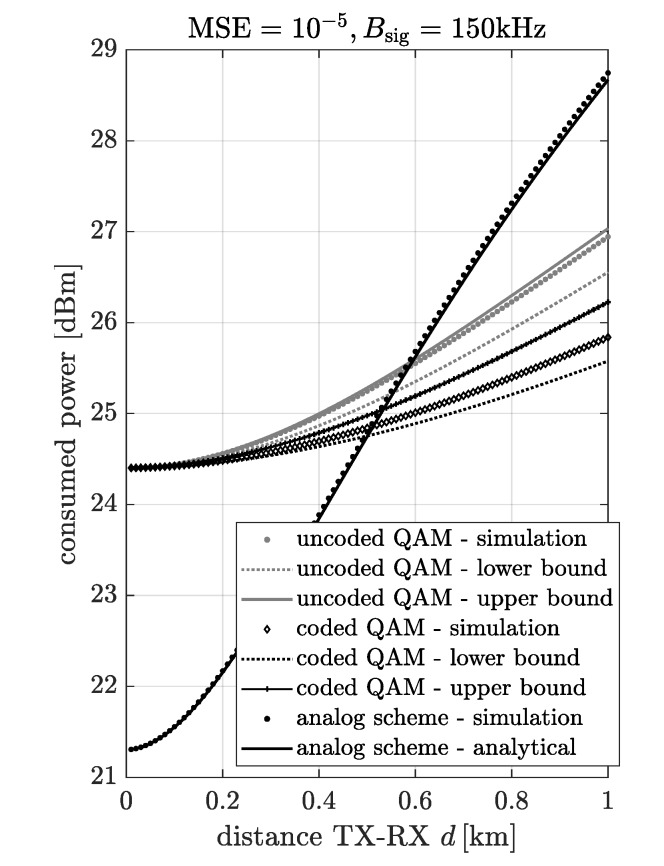
The power consumption of the digital and analog scheme resulting from the simulation and analytical model vs. distance between transmitter and receiver for $\mathrm{MSE}=10^{-5}$, $B_{\mathrm{sig}}=150\;\mathrm{kHz}$ and $\zeta_{\mathrm{FM}}=0.5$.

In Figure 10, the power consumption of the digital and analog scheme resulting from the simulation and analytical model vs. distance between transmitter and receiver for $\mathrm{MSE}=10^{-5}$, $B_{\mathrm{sig}}=150\;\mathrm{kHz}$ and $\zeta_{\mathrm{FM}}=0.5$ is presented. In the case of the digital scheme, the lower and upper bound of the power consumption are plotted. It can be observed that the simulation results are between curves obtained by the upper and lower bound. Moreover, in the case of the analog scheme, the approximation fits to the simulation results very well.

## 5. Conclusions

In this paper, inspired by the human brain, we have analyzed the analog and digital transmission from the energy-efficiency point of view. Our motivation was to flexibly choose between both schemes in a massive, short-links network to transfer information from various sources to various sinks, over moderately reliable channels with various QoS requirements. In our paper, we assumed that the transmitted data came from analog sensors in all systems. Therefore, in the case of the digital transmission, the input data are fed first to the analog-to-digital converter. At the receiving side, the decoded data are converted back to analog form. The following transmission schemes have been compared in the context of the performance as well as of the energy efficiency: simple analog, simple digital uncoded ASK and more complex digital uncoded and coded QAM scheme. For the energy efficiency analysis, the circuit power consumption models as well as datasheets have been studied and included in the total power consumption model. The high-level power consumption model and the relation factor have been applied in order to make the results independent from technologies. It can be observed that the power consumed when the QAM transmitter and receiver are turned on is constant and $P_{\mathrm{CD}}^{\mathrm{ASK}}$ and $P_{\mathrm{CA}}$ depend on this value, directly. Therefore, the change of this value causes the dilution of the power consumption about the difference between this value and the new value while the relations between transmission schemes will be the same. The simulation results have been obtained for the same bandwidth and the same SNR in the occupied band and for the same MSE at the receiver output (related to the system performance). They show that in some cases, especially for relatively short links, the analog transmission can be beneficial, resulting in higher energy efficiency, while assuming the same performance level. Furthermore, the difference between energy consumption of frequency modulation and a simple digital modulation (ASK) is small and implementation dependent. Therefore, the proposed model gains importance providing not only qualitative but also quantitative guidance for energy efficient transmission mode selection. Moreover, due to the fact that the most data is represented in the digital form (sources are discrete), the simple uncoded digital communication system (with ASK modulation) has also been compared with another digital system with QAM modulation. The results show that there exist cases when the simple scheme minimizes the consumed power. Moreover, there exists optimal bandwidth extension which minimizes the power consumption for the considered analog and digital communication systems. Finally, the analytical power consumption model of the considered schemes has been proposed. The results show that the analytical model fits the simulation data very well and can be used to design the adaptive selection between analog and digital scheme in the link or in the multiuser network. In the case of multiuser interference, the proposed power consumption model can be easily adapted because in many cases for the considered narrowband system, the interference can be modeled as an increased noise floor. Therefore, the performance in the interference-limited environment can be estimated by calculating SINR value and using it for an SNR value in the presented results.

## Figures and Tables

**Table 1:** Power consumption of FM chips.

Name	Description	Typ. [mW]	Max. [mW]
Niigata Seimitsu Co. NS73M-61LU	FM transmitter, full CMOS process	108.0	135.0
ON Semiconductor LA72914V	FM modulator and demodulator	165.0	200.0
New Japan Radio Co. NJM2519A	FM Modulator for VHF Band	76.5	95.1
RDA Micoelectronics RDA5820	single-chip broadcast FM, CMOS process	63.4	74.3
Atmel U4065B	FM Receiver	296.0	376.0
Philips TDA1596T	FM Demodulator	170.0	221.0
Philips TDA7088T	FM receiver circuit for battery supply	15.6	19.8
Silicon Labs Si4702/03-C19	Broadcast FM radio tuner for portable applications	165.0	181.5
	**Average**	132.4	162.8

**Table 2:** The fitting parameters for the analog transmission scheme.

Analog Scheme	$a^{\mathrm{FM}}$		$b^{\mathrm{FM}}$		$c^{\mathrm{FM}}$	$d^{\mathrm{FM}}$
$\mathrm{MSE}=10^{-4}$	52.18		−0.4481		11.20	−0.001007
$\mathrm{MSE}=10^{-5}$	43.78		−0.2342		13.32	0.000255

**Table 3:** The fitting parameters for the coded digital transmission scheme.

Coded QAM	$a_{\mathrm{coded}}^{\mathrm{QAM}}$		$b_{\mathrm{coded}}^{\mathrm{QAM}}$		$c_{\mathrm{coded}}^{\mathrm{QAM}}$		$d_{\mathrm{coded}}^{\mathrm{QAM}}$	
	lower	upper	lower	upper	lower	upper	lower	upper
$\mathrm{MSE}=10^{-4}$	39.04	45.01	−0.5964	−0.3550	26.43	22.38	−0.1168	−0.07004
$\mathrm{MSE}=10^{-5}$	30.85	46.03	−0.1491	−0.4444	1.931	28.02	−0.02527	−0.07516

**Table 4:** The fitting parameters for the uncoded digital transmission scheme.

Uncoded QAM	$a_{\mathrm{uncoded}}^{\mathrm{QAM}}$		$b_{\mathrm{uncoded}}^{\mathrm{QAM}}$		$c_{\mathrm{uncoded}}^{\mathrm{QAM}}$	
	lower	upper	lower	upper	lower	upper
$\mathrm{MSE}=10^{-4}$	50.42	90.86	−1.037	−1.176	8.575	9.43
$\mathrm{MSE}=10^{-5}$	49.88	88.68	−1.01	−1.162	9.144	10.32

## Abbreviations

The following abbreviations are used in this manuscript:

ADC	Analog to Digital Converter
AI	Artificial Intelligence
ASK	Amplitude-Shift Keying
DAC	Digital to Analog Converter
FEC	Forward Error Correction
FM	Frequency Modulation
IFA	Intermediate Frequency Amplifier
IoT	Internet of Things
MSE	Mean Square Error
PA	Power Amplifier
PAR	Peak to Average Power Ratio
PLL	Phase Locked Loop
QAM	Quadrature Amplitude Modulation
RFA	Radio Frequency Amplifier
SNR	Signal to Noise Ratio
VCO	Voltage-Controlled Oscillator

## References

[1] Cisco Visual Networking Index: Global Mobile Data Traffic Forecast Update. http://s3.amazonaws.com/media.mediapost.com/uploads/CiscoForecast.pdf.

[2] Cisco Annual Internet Report (2018–2023) White Paper. http://www.cisco.com/c/en/us/solutions/collateral/executive-perspectives/annual-internet-report/white-paper-c11-741490.html.

[3] Cui S.G., Goldsmith A.J., Bahai A. (2004). Energy-efficiency of MIMO and cooperative MIMO techniques in sensor networks. IEEE J. Sel. Areas Commun..

[4] Kryszkiewicz P., Kliks A. Modeling of Power Consumption by Wireless Transceivers for System Level Simulations. Proceedings of the European Wireless 2017, 23th European Wireless Conference.

[5] Dressler F., Akan O. (2010). A Survey on Bio-inspired Networking. Comput. Netw..

[6] Meisel M., Pappas V., Zhang L. (2010). A Taxonomy of Biologically Inspired Research in Computer Networking. Comput. Netw..

[7] Zhirnov V.V., Cavin R.K. (2013). Future Microsystems for Information Processing: Limits and Lessons From the Living Systems. IEEE J. Electron Devices Soc..

[8] Nakano T. (2011). Biologically Inspired Network Systems: A Review and Future Prospects. IEEE Trans. Syst. Man Cybern. Part C Appl. Rev..

[9] Bullmore E., Sporns O. (2012). The economy of brain network organization. Nat. Rev. Neurosci..

[10] Khozeimeh F., Haykin S. (2012). Brain-Inspired Dynamic Spectrum Management for Cognitive Radio Ad Hoc Networks. IEEE Trans. Wireless Commun..

[11] Li R., Zhao Z., Zhou X., Ding G., Chen Y., Wang Z., Zhang H. (2017). Intelligent 5G: When Cellular Networks Meet Artificial Intelligence. IEEE Wirel. Commun..

[12] Wang X., Li X., Leung V.C.M. (2015). Artificial Intelligence-Based Techniques for Emerging Heterogeneous Network: State of the Arts, Opportunities, and Challenges. IEEE Access.

[13] Kliks A., Kulacz L. Brain Inspirations for Dense Wireless Networks: Microglia Functionality. Proceedings of the 2018 IEEE 29th Annual International Symposium on Personal, Indoor and Mobile Radio Communications (PIMRC).

[14] Mezghani A., Nossek J.A. Power efficiency in communication systems from a circuit perspective. Proceedings of the 2011 IEEE International Symposium of Circuits and Systems (ISCAS), Rio de Janeiro.

[15] Haykin S. (2009). Communication Systems.

[16] Li Y., Bakkaloglu B., Chakrabarti C. (2007). A System Level Energy Model and Energy-Quality Evaluation for Integrated Transceiver Front-Ends. IEEE Trans. Very Large Scale Integr. VLSI Syst..

[17] Duarte D., Vijaykrishnan N., Irwin M.J. A complete phase-locked loop power consumption model. Proceedings of the 2002 Design, Automation and Test in Europe Conference and Exhibition.

[18] Lauwers E., Gielen G. (2002). Power estimation methods for analog circuits for architectural exploration of integrated systems. IEEE Trans. Very Large Scale Integr. VLSI Syst..

[19] Ochiai H. (2013). An Analysis of Band-limited Communication Systems from Amplifier Efficiency and Distortion Perspective. IEEE Trans. Commun..

[20] Isheden C., Fettweis G. Energy-Efficient Multi-Carrier Link Adaptation with Sum Rate-Dependent Circuit Power. Proceedings of the 2010 IEEE Global Telecommunications Conference GLOBECOM 2010.

[21] Isheden C., Fettweis G. Energy-Efficient Link Adaptation with Transmitter CSI. Proceedings of the 2011 IEEE Wireless Communications and Networking Conference.

[22] Lu Y., Xiong K., Zhang Y., Fan P., Zhong Z. Energy-Efficient Resource Allocation in OFDM Relay Networks under Proportional Rate Constraints. Proceedings of the 2016 IEEE Global Communications Conference (GLOBECOM).

[23] Xiong K., Fan P., Lu Y., Letaief K.B. (2016). Energy Efficiency With Proportional Rate Fairness in Multirelay OFDM Networks. IEEE JSAC.

[24] Kryszkiewicz P., Idzikowski F., Bossy B., Kopras B., Bogucka H. Energy Savings by Task Offloading to a Fog Considering Radio Front-End Characteristics. Proceedings of the 2019 IEEE 30th Annual International Symposium on Personal, Indoor and Mobile Radio Communications (PIMRC).

[25] Björnson E., Sanguinetti L., Hoydis J., Debbah M. (2015). Optimal Design of Energy-Efficient Multi-User MIMO Systems: Is Massive MIMO the Answer?. IEEE Trans. Wireless Commun..

[26] ETSI LTE; Evolved Universal Terrestrial Radio Access (E-UTRA); Radio Frequency (RF) System Scenarios (3GPP TR 36.942 version 10.2.0 Release 10), Annex C, Table C.1. https://www.etsi.org/deliver/etsi_tr/136900_136999/136942/10.02.00_60/tr_136942v100200p.pdf.

[27] Lathi B.P. (1995). Modern Digital and Analog Communication Systems.

